# Desloratadine, an FDA-approved cationic amphiphilic drug, inhibits SARS-CoV-2 infection in cell culture and primary human nasal epithelial cells by blocking viral entry

**DOI:** 10.1038/s41598-022-25399-5

**Published:** 2022-12-06

**Authors:** Margot Morin-Dewaele, Sophie Bartier, François Berry, Rozenn Brillet, Dennis Salomón López-Molina, Công Trung Nguyễn, Pascale Maille, Kevin Sereno, Quentin Nevers, Laurent Softic, Jean-Marie Vaugeois, Bruno Louis, Emilie Bequignon, Patrice Bruscella, André Coste, Jean-Michel Pawlotsky, Stéphane Jamain, Abdelhakim Ahmed-Belkacem

**Affiliations:** 1 Univ Paris Est Créteil, INSERM U955, IMRB, Créteil, France; 2 Service d’ORL et de Chirurgie Cervico-Faciale, Centre Hospitalier Intercommunal de Créteil, 94000 Créteil, France; 3 Service d’ORL et de Chirurgie Cervico-Faciale, AP-HP, Centre Hospitalier Universitaire Henri Mondor, 94000 Créteil, France; 4 CNRS, ERL 7000, 94000 Créteil, France; 5 Department of Pathology, University Hospital Henri Mondor, AP-HP, Créteil, France; 6 Institute for Integrative Biology of the Cell (I2BC), CEA, CNRS, Université Paris-Saclay, Gif-Sur-Yvette, France; 7 Université de Paris, Institut Cochin, INSERM, CNRS, 75014 PARIS, France; 8 Normandie Univ, UNIROUEN, UNICAEN, ABTE, 76000 Rouen, France; 9 Department of Virology, Hôpital Henri Mondor, AP-HP, Université Paris-Est, Créteil, France; 10 Translational Neuropsychiatry, Fondation FondaMental, 94010 Créteil, France

**Keywords:** Antivirals, Viral infection

## Abstract

The 2019 global coronavirus (COVID-19) pandemic has brought the world to a grinding halt, highlighting the urgent need for therapeutic and preventive solutions to slow the spread of emerging viruses. The objective of this study was to assess the anti-SARS-CoV-2 effectiveness of 8 FDA-approved cationic amphiphilic drugs (CADs). SARS-CoV-2-infected Vero cells, Calu-3 cells and primary Human Nasal Epithelial Cells (HNEC) were used to investigate the effects of CADs and revealed their antiviral mode of action. Among the CADs tested, desloratadine, a commonly used antiallergic, well-tolerated with no major side effects, potently reduced the production of SARS-CoV-2 RNA in Vero-E6 cells. Interestingly, desloratadine was also effective against HCoV-229E and HCoV-OC43 showing that it possessed broad-spectrum anti-coronavirus activity. Investigation of its mode of action revealed that it targeted an early step of virus lifecycle and blocked SARS-CoV-2 entry through the endosomal pathway. Finally, the ex vivo kinetic of the antiviral effect of desloratadine was evaluated on primary Human Nasal Epithelial Cells (HNEC), showing a significant delay of viral RNA production with a maximal reduction reached after 72 h of treatment. Thus, this treatment could provide a substantial contribution to prophylaxis and systemic therapy of COVID-19 or other coronaviruses infections and requires further studies.

## Introduction

Coronaviruses are a group of enveloped positive-sense RNA viruses. Until recently, most human infections were caused by four human coronaviruses (HCoV) inducing benign respiratory tract diseases, including HCoV-OC43, HCoV-229E, HCoV-NL63 and HKU1. In the last two decades, three zoonotic coronaviruses capable to induce severe lung disease with moderate to high lethality rates have emerged in human populations. The Severe Acute Respiratory Syndrome Coronavirus (SARS-CoV) emerged in 2003 in China and caused a self-limiting outbreak^1^. In 2012, the Middle East Respiratory Syndrome Coronavirus (MERS-CoV) emerged in Saudi Arabia. This virus is rarely transmitted between humans, but has a very high lethality rate^2^. Lastly, the SARS-CoV-2, a hitherto unknown member of the *Orthocoronavirinae* subfamily, emerged in december 2019 in China and rapidly spread worldwide, causing the still-ongoing Coronavirus Disease 2019 (COVID-19) pandemic^3^. As of November 3, 2021, the World Health Organization reported a total of 248,466,602 cases of SARS-CoV-2 infection, and 5,032,252 deaths (https://www.worldometers.info/coronavirus/). Vaccine development has been successful in preventing severe disease and mortality in Western countries. However, this protection strategy raises concerns about the difficulty of vaccinating the entire global population, particularly in developing countries, and the high mutation frequencies of the SARS-Cov-2 proteins targeted by the vaccines. At the beginning of the pandemic, only nonspecific treatments were available, but recently, in addition to Remdesivir, Molnupiravir and Nirmatrelvir, two oral antivirals targeting the viral polymerase and the main protease of SARS-CoV-2, respectively, received emergency use approval from the U. S. Food and Drug Administration for the treatment of mild-to-moderate COVID-19. Nevertheless, Molnupiravir and Nirmatrelvir present some issues regarding potential toxicity in children and pregnant women and potential drug-drug interactions, respectively, precluding their use in some at-risk patients. Therefore, there is a need to develop other oral drugs that do not have the limitations of these drugs for more widespread use.

Coronaviruses have demonstrated their high capacity to cross species barriers from animal reservoirs to human populations. A large number of coronaviruses have been identified to share this capacity, notably SARS-like viruses circulating in Chinese bat populations^4,5^. Therefore, they represent an ongoing threat to the global public health and economy, highlighting the urgent need for the development of active broad-spectrum antiviral drugs that can be used in the context of various viral emergences, without having to wait for the availability of specifically designed antiviral drugs.

Cationic amphiphilic drugs (CADs) include hundreds of FDA-approved agents used to treat a broad spectrum of common diseases, including psychiatric disorders, allergies, heart diseases and infections^6^. CADs are characterized by a hydrophobic aromatic ring or ring system and a hydrophilic side chain containing an ionizable amine functional group^7^. CADs are lysosomotropic drugs as they accumulate into acidic compartments, such as late endosomes/lysosomes, leading to an impairment of lysosomal functions^8–10^. Because the endosome/lysosome is considered as a key cell organelle involved in virus entry and egress, CADs have gained increasing attention as candidate drugs for repurposing^11–14^. SARS-CoV-2 entry requires cleavage of the Spike envelope glycoprotein (S) by host cell proteases. Depending on cell protease availability, cleavage can occur at the cell surface mediated by Transmembrane Serine Protease 2 (TMPRSS2)^15^ or be catalyzed by endosome-residing proteases Cathepsins L and B (CatL and CatB)^16,17^. Both pathways have been shown to contribute to SARS-CoV-2 infection in vitro, depending on the cellular model used and its natural equipment^15^.

In this study, we explored the effect of 8 FDA-approved CADs on SARS-CoV-2 replication in vitro in Vero-E6 cells. Among them, desloratadine, a commonly used antiallergic compound^18^, was highly effective in reducing SARS-CoV-2 RNA production in Vero-E6 cells. Further characterization of desloratadine antiviral properties included evaluation of its anti-coronavirus spectrum, identification of its target viral step and investigation of its antiviral mechanism of action, was performed. Finally, we used an ex vivo model of infection with SARS-CoV-2, primary Human Nasal Epithelial Cells (HNECs) from different donors, to measure the antiviral efficacy of desloratadine. Our results suggest that repurposing of desloratadine could be proposed in SARS-CoV-2 infection and early after the future emergence of new coronaviruses, with well-known, limited and easily manageable side effects and therefore requires further in *vivo* investigations.

## Materials and methods

### Cells and viruses

Vero-E6 cells (ATCC CRL-1586) and MRC-5 cells (ATCC, CCL-171) were maintained in Dulbecco’s modified Eagle medium (DMEM; ThermoFischer Scientific, Waltham, MA, USA) supplemented with 50 IU/mL of penicillin, 100 µg/mL of streptomycin, 10% of fetal bovine serum (FBS) and 0.1 µg/mL of fungizone (ThermoFischer Scientific). Calu-3 cells (ATCC, HTB-55) and HRT18 cells (ATCC, CCL-244) were maintained in the same media supplemented with non-essential amino acids (ThermoFischer Scientific) and 10% of sodium bicarbonate (Gibco™), respectively. Primary HNECs were obtained from nasal polyps (NP) from patients with chronic rhinosinusitis undergoing ethmoidectomy, as previously described^19^ and cultivated at the air–liquid interface (ALI). HNECs reach a stable differentiated state with the detection of ciliated, secretory, and basal cells during the third week of culture^20^.

Primary HNECs from a pool of 14 different healthy donors were obtained commercially from Ephitelix (Epithelix Sarl, Geneva, Switzerland) and cultured in MucilAir™ culture medium (Epithelix Sarl, Geneva, Switzerland) according to the manufacturer’s instructions. SARS-CoV-2 (variant of origin D614G) was isolated from nasopharyngeal swabs of a symptomatic patient infected during the first French epidemic wave and amplified by passages in Vero-E- cells. HCoV-229E and OC-43 strains were kindly provided by Pr. Astrid Vabret (University of Caen, Caen, France).

### Compounds

Compounds were obtained from ThermoFisher Scientific (amitriptyline hydrochloride, carbamazepine) and Sigma-Aldrich (chlorpromazine hydrochloride, chlorpromazine sulfoxide, clemastine fumarate salt, desloratadine, haloperidol, imipramine hydrochloride, loratadine, remdesivir, terfenadine, camostat mesylate and E64D). All compounds were solubilized in dimethyl sulfoxide (DMSO). The final concentration of DMSO was 0.01% for all experiments.

### Assessment of antiviral activity

Vero-E6 cells and Calu3 cells were infected for 2 h with SARS-CoV-2 at a Multiplicity Of Infection (MOI) of 0.2 and 0.5 in the presence or in the absence of the tested compounds. After 48 h, SARS-CoV-2 RNA was extracted from cells or cell supernatants and quantified by RT-qPCR using Taqman technology. SARS-CoV-2 RNA relative quantities were plotted against compound concentrations and fitted with a four-parameter logistic curve with SigmaPlot v11 software. The effective concentrations 50% (EC_50_) were determined from the curves. MRC5 cells and HRT-18 cells were infected at an MOI of 0.5 for 2 h with HCoV-229E and HCoV-OC43, respectively. After 24 h, viral RNA was extracted from cells using QIAamp Viral RNA Mini kit” (Qiagen) and quantified by RT-qPCR. The data were analyzed with the 2^−ΔΔCt^ method, with values for all samples normalized to the value for glyceraldehyde-3-phosphate dehydrogenase (GAPDH) for intracellular samples.

### Immunofluorescence staining

Immunofluorescence staining of Vero-E6 cells was performed using an antibody directed against double-stranded RNA (dsRNA), as previously described^21^. Infected cells were quantified using ImageJ software (National Institutes of Health, Bethesda, Maryland, USA).

### Time-of-addition assay

Vero-E6 cells were seeded in a 48-well plate at a density of 7.5 × 10^4^ cells/well and incubated for 24 h at 37 °C in 5% CO_2_. Cells were infected with SARS-CoV-2 for 2 h at an MOI of 1. Ten µM of the indicated compound was added 2 h prior to infection, at the time of infection or 3, 6, 9 or 12 h post-infection and SARS-CoV-2 RNA production was measured 24 h post-infection by means of RT-qPCR.

### Effect of the compounds on the time course of HNEC infection

Twenty µL of SARS-CoV-2 inoculum with a Median Tissue Culture Infectious Dose of 5 × 10^4^/mL (TCID_50_) was added at the apical pole of the epithelium in the presence of various concentrations of the compounds. After 4 h, the viral inoculum was removed and cells were washed with 200 µL of PBS before addition of 20 µL of the different concentrations of compounds. To collect viral RNA produced, the apical pole of the epithelium was washed with 200 µl of PBS for 10 min and SARS-CoV-2 RNA was extracted using QIAamp Viral RNA Mini kit” (Qiagen) and measured 4, 24, 48, 72, and 96 h post-infection by RT-qPCR. Epithelial integrity was assessed by measuring transepithelial electrical resistance (TEER) at the same time using an EVOM volt-ohmmeter (World Precision Instruments, Sarasota, FL, USA).

### Immunohistochemistry staining of HNECs

After 4 weeks of culture, HNEC epithelia were removed from transwells and fixed in 10% formalin for 12 h. Each membrane was embedded vertically into a paraffin block. Paraffin-embedded blocks were then cut into 4 µm‐thick slides. Sections were deparaffinised, rehydrated and treated for antigen retrieval with citrate buffer. After blocking for 1 h using BSA 5%, sections were incubated with primary antibodies targeting SARS-CoV-2 nucleoprotein (Genetex 135357) or α-tubuline (Abcam 24610) and secondary antibody (Alexa Fluor) for 90 min at room temperature.

### Statistical analyses

The experiments were performed in triplicate. Data are expressed as mean ± SEM or percentages. Statistical differences between the means of two datasets were assessed by means of the Mann‐Whitney U‐test using Graphpad Prism software. Asterisks indicate p-values as *: *p* < 0.05, **: *p* < 0.01, NS not significantly different.

## Results

### In vitro screening of FDA-approved CAD effectiveness against SARS-CoV-2 RNA production

**Figure 1:**
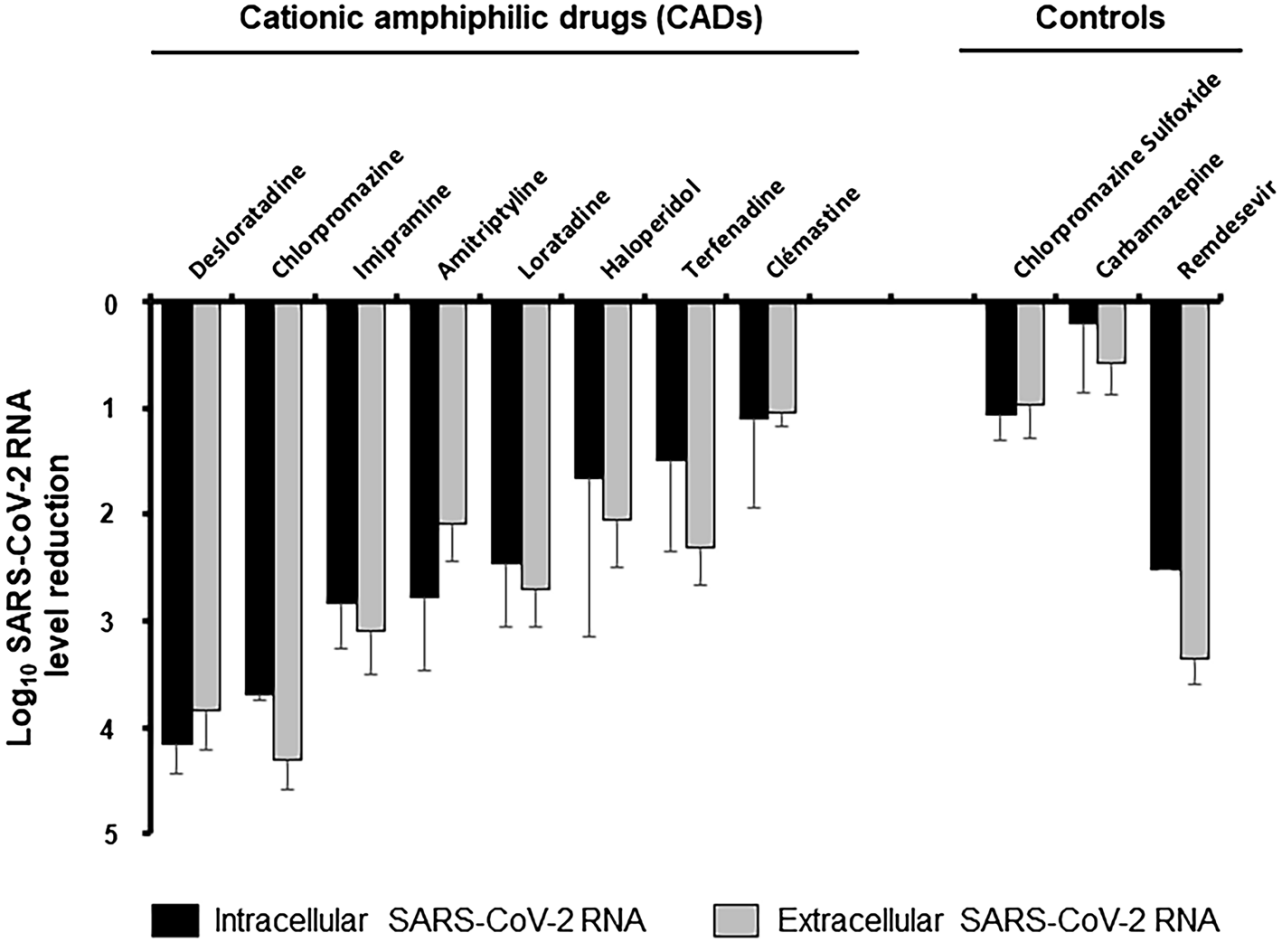
Antiviral effect of 10 µM of FDA-approved CADs on SARS-CoV-2 RNA production in Vero-E6 cells. Vero-E6 cells were treated with a fixed dose of 10 µM of the different CADs and infected with SARS-CoV-2 at an MOI of 0.2. Forty-eight hours post-infection, viral RNA was extracted from cells and cell supernatants and quantified by RT-qPCR. Intracellular (black bar) and extracellular (grey bar) SARS-CoV-2 RNA log decreases are shown related to untreated controls. Each bar represents the mean ± SD of at least two independent experiments performed in triplicate.

Eight FDA-approved CADs (Supplementary Fig. 1) were tested for their ability to reduce intracellular and extracellular SARS-CoV-2 RNA production at a fixed dose of 10 µM in Vero-E6 cells (Fig. 1). Carbamazepine^22^ and remdesivir were used as negative and positive controls, respectively. Carbamazepine shares a chemical scaffold similar to that of some of the compounds tested, but it is not a CAD because it does not contain an ionizable amine. Remdesivir is an RNA-dependent RNA polymerase inhibitor that has been reported to potently inhibit SARS-CoV-2 replication in vitro^23^.

Five CADs with distinct pharmacological targets significantly reduced SARS-CoV-2 RNA production (> 2-log reduction) in both Vero-E6 cell extracts and supernatants, suggesting that their antiviral effect was not related to their main pharmacological target, but rather to their common physicochemical properties. Three CADs displayed intermediate antiviral effectiveness, with viral RNA level reductions ranging from 1- to 2-log at 10 µM. As expected, carbamazepine had no effect on SARS-CoV-2 RNA production, whereas remdesivir potently inhibited it.

Structure–activity relationship analysis revealed that the 5 most potent compounds all contained a tricyclic ring skeleton (Supplementary Fig. 1), which thus appears to be an important chemical determinant for CAD antiviral activity. To confirm this hypothesis, we evaluated the effect of chlorpromazine sulfoxide, which differs from chlorpromazine only by the addition of a sulfoxide moiety on the tricyclic ring. The antiviral potency of chlorpromazine sulfoxide was drastically reduced as compared to that of chlorpromazine (Fig. 1), confirming that the modification of the tricyclic ring was detrimental to the compound’s antiviral effectiveness.

Together, these results strongly suggest that tricyclic CADs are a class of compounds with antiviral activity against SARS-CoV-2, that provide a promising scaffold for the development of antiviral compounds.

### Characterization of CAD in vitro anti-SARS-CoV-2 activity

**Figure 2:**
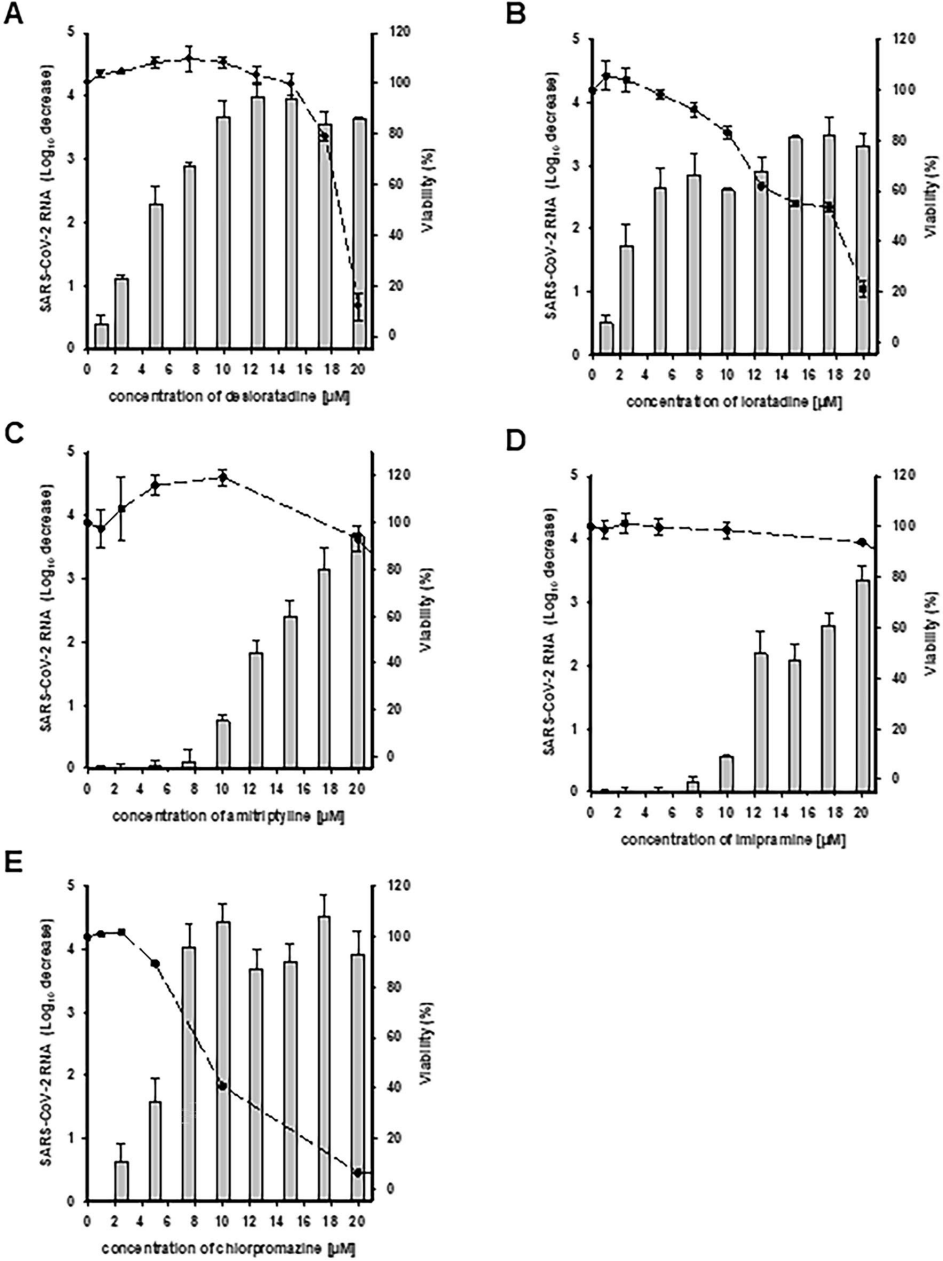
Assessment of the dose-dependent antiviral effectiveness of CADs against SARS-CoV-2 in Vero-E6 cells. Vero-E6 cells were infected with SARS-CoV-2 at an MOI of 0.2 in the presence of increasing concentrations of: (**A**) desloratadine, (**B**) loratadine, (**C**) amitriptyline, (**D**) imipramine and (**E**) chlorpromazine. The reduction of the extracellular production of SARS-CoV-2 RNA (grey bars) was quantified by RT-qPCR and expressed relative to the untreated control. Vero-E6 viability (black points) was monitored by MTS assay. Each bar and point represents the mean ± SD of at least two independent experiments performed in triplicate.

The anti-SARS-CoV-2 effectiveness and the cytotoxicity of increasing concentrations of the 5 most potent compounds identified in the previous step were measured in Vero-E6 cells (Fig. 2). All compounds induced a dose-dependent decrease of SARS-CoV-2 RNA production. The antihistaminic compounds desloratadine and loratadine were the most potent ones, with EC_50_s of 0.7 ± 0.4 µM and 0.9 ± 0.3 µM, respectively. These two compounds were not cytotoxic at their effective concentrations with CC_50_s of 15.5 ± 1.1 and 19.9 ± 4.5 µM and therapeutic indexes of 23.3 and 22.5, respectively (Table 1). Loratadine and desloratadine inhibited SARS-CoV-2 RNA extracellular production up to a maximum of 2265-fold and 6673-fold, respectively. Desloratadine was selected for further antiviral characterization.

**Table 1:** EC_50_s, CC_50_s and therapeutic indexes of the 5 most potent CADs tested.

	EC_50_ of SARS-CoV-2 RNA production inhibition (µM)	CC_50_ in Vero-E6 cells (µM)	Therapeutic index
Loratadine	0.7 ± 0.4	15.5 ± 1.1	23.3
Desloratadine	0.9 ± 0.3	19.9 ± 4.5	22.5
Amitriptyline	8.8 ± 1.4	25.2 ± 1.7	2.9
Imipramine	8.1 ± 0.4	32.8 ± 1.0	4.1
Chlorpromazine	3.6 ± 1.2	8.7 ± 0.2	2.4

### Spectrum of desloratadine antiviral activity against different coronaviruses

**Figure 3:**
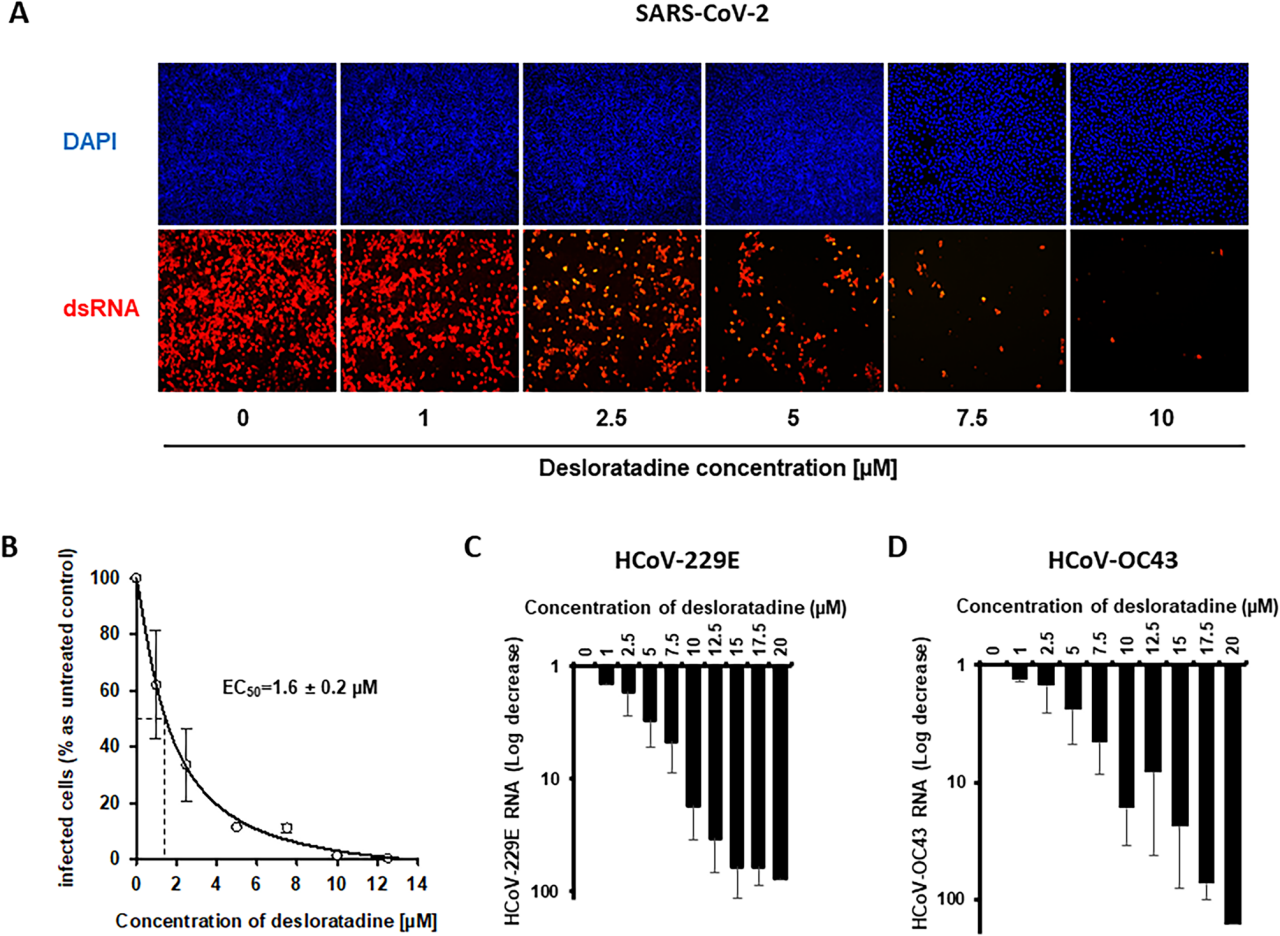
Assessment of desloratadine antiviral activity against SARS-CoV-2, HCoV-229E and HCoV-OC43. Vero-E6, MRC-5 and HRT-18 cells were infected with SARS-CoV-2 (MOI = 0.2), HCoV-229E (MOI = 0.5) and HCoV-OC43 (MOI = 0.5), respectively, and treated with increasing concentrations of desloratadine. (**A**) SARS-CoV-2 infection of Vero-E6 cells monitored by immunofluorescence with an antibody directed against double stranded RNA (dsRNA). (**B**) SARS-CoV-2-infected Vero-E6 cells quantified by image J software. Effect of increasing concentrations of desloratadine on HCoV-229 (**C**) and HCoV-OC43 (**D**) viral RNA production in MRC-5 and HRT-18 cell supernatants, respectively, quantified by RT-qPCR.

The effect of desloratadine on SARS-CoV-2 infection was measured in Vero-E6 cells by means of immunofluorescence using an antibody directed against double-stranded RNAs. A dose-dependent decrease of the number of SARS-CoV-2 infected cells was observed (Fig. 3A) and quantified (Fig. 3B). The EC_50_ of desloratadine on SARS-CoV-2 infection was 1.6 ± 0.2 µM.

In order to characterize the spectrum of antiviral effectiveness of desloratadine, its effect on RNA production from two other benign coronaviruses, HCoV-229E and HCoV-OC43, was measured by RT-qPCR in MRC5 and HRT-18 cells, respectively (Fig. 3C,D). Desloratadine induced a dose-dependent decrease of viral RNA production in both models. These results indicate that desloratadine carries broad-spectrum antiviral effectiveness against coronaviruses, suggesting a common mechanism of action against this viral family.

### Identification of the SARS-CoV-2 lifecycle step targeted by desloratadine

**Figure 4:**
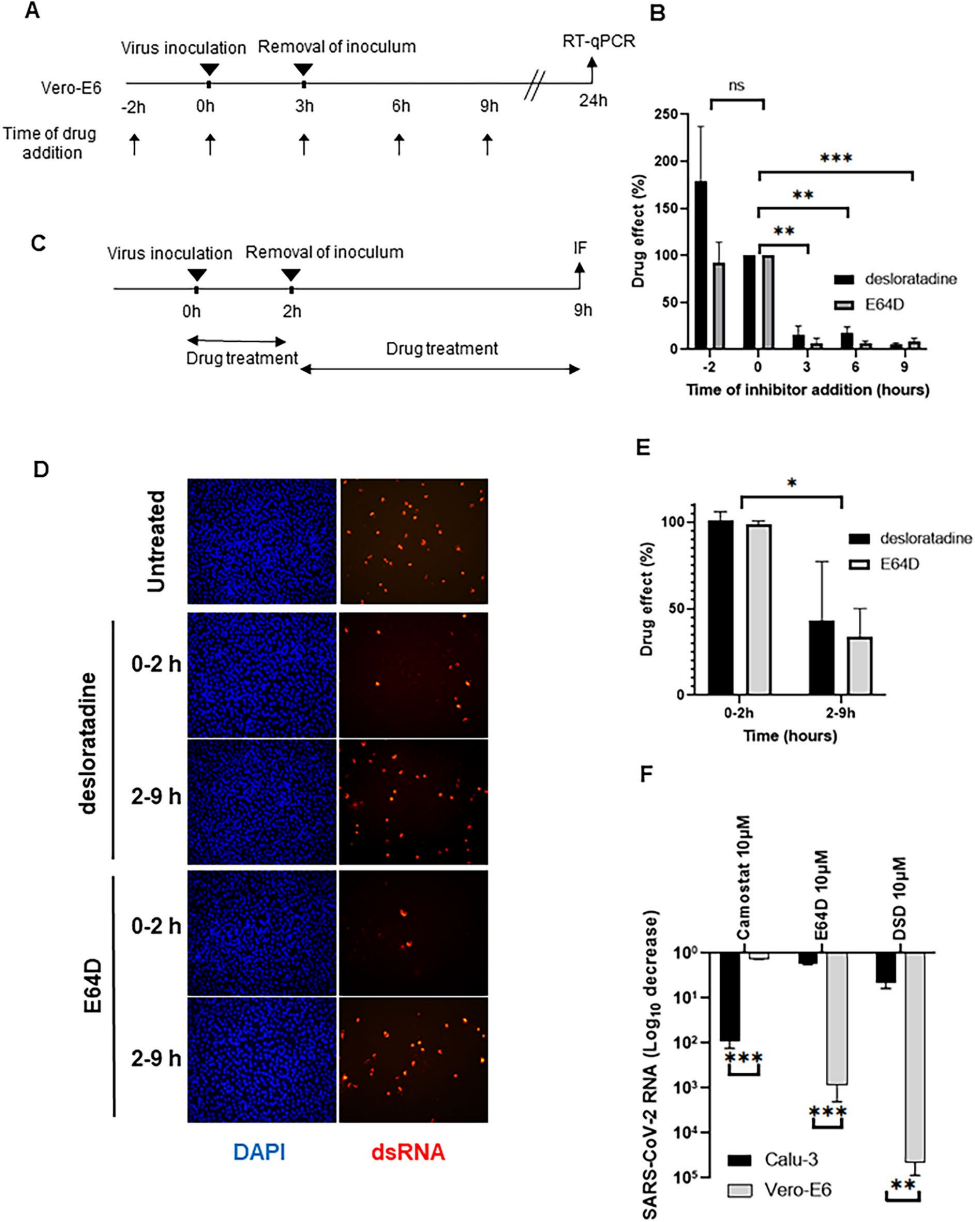
Desloratine inhibition of SARS-CoV-2 infection at an early step of the viral lifecycle. (**A**) Design of the time-of-drug addition assay in Vero-E6 cells. (**B**) Results of the time-of-drug addition assay using 10 µM of desloratadine (black bar) or 10 µM of E64D (grey bar), expressed as the percent effect of the drug at each time point relative to its effect when added at the time of infection, considered as 100%. (**C**) Design of the entry assay using desloratadine and E64D. 10 µM of the indicated drugs were added either during the first 2 h of infection or after removal of the inoculum between the 2nd and the 9th hours. (**D**) Cells infected at different time points studied by immunofluorescence using an antibody directed against double-stranded RNA (dsRNA). (**E**) Quantification of immunofluorescence in cells infected at different time points. The percent effect of 10 µM of desloratadine (black bars) and 10 µM of E64D (grey bars) is shown relative to its effect when the drugs were added between 0 and 2 h, considered as 100%. (**F**) Effect of 10 µM of desloratadine, camostat and E64D on SARS-CoV-2 RNA production in Vero-E6 (grey bars) and TMPRSS2-expressing Calu-3 cells (black bars), as assessed by RT-qPCR.

Using 10 µM for each drugs, a time-of-addition assay was performed in Vero-E6 cells as described in Fig. 4A. Aloxistatin (E64D), a cell-permeable and irreversible broad-spectrum cysteine protease inhibitor known to block coronavirus lifecycles at the entry step, was used as a control. Both desloratadine and E64D antiviral effects were drastically impaired when the compounds were added 3 h post-infection (Fig. 4B). This result indicated that desloratadine does not block the SARS-CoV-2 lifecycle at a post-entry step.

To confirm that desloratadine targets an early step of the SARS-CoV-2 lifecycle, we compared its antiviral effect at 10 µM when added during *vs* after the first 2 h of infection, as described in Fig. 4C. The amount of SARS-CoV-2-infected cells was reduced only when desloratadine was present during the first 2 h of infection (Figs. 4D). A similar result was obtained with the control entry inhibitor E64D used at 10 µM. Quantification of immunofluorescence staining showed that both desloratadine and E64D lost more than 50% of their antiviral effectiveness when added more than 2 h after infection (Fig. 4E). These results demonstrate that desloratadine targets the entry step of SARS-CoV-2 lifecycle in Vero-E6 cells.

### Identification of the SARS-CoV-2 entry mode targeted by desloratadine

Depending on the availability of host proteases capable to prime the spike protein, SARS-CoV-2 can deliver its RNA into its target cells through fusion of its envelope either with the plasma membrane or the endosome membrane. Therefore, the effect of 10 µM of desloratadine was evaluated in Vero-E6 and in Calu-3 cells, cell lines in which SARS-CoV-2 has been described to mainly fusion at the endosome membrane level and at the plasma membrane level, respectively^24^ (Fig. 4F). 10 µM of E64D and camostat were used as controls. E64D alters viral fusion at the endosome membrane through inhibition of the endosome-resident cysteine proteases cathepsins, whereas camostat alters viral fusion at the plasma membrane by inhibiting the cell surface serine protease TMPRSS2. As expected, camostat significantly inhibited SARS-CoV-2 infection in Calu-3 cells, whereas it was less effective in Vero-E6 cells. Conversely, E64D effectively reduced SARS-CoV-2 infection in Vero-E6 cells, whereas no significant inhibition was observed in Calu-3 cells with this compound. The effect of desloratadine was similar to that of E64D, i.e. potently inhibitory against SARS-CoV-2 infection in Vero-E6 cells, but not in Calu-3 cells. These findings indicate that desloratadine blocks SARS-CoV-2 entry via the endosomal route.

### Assessment of desloratadine anti-SARS-CoV-2 activity in primary human nasal epithelial cells (HNEC)

**Figure 5:**
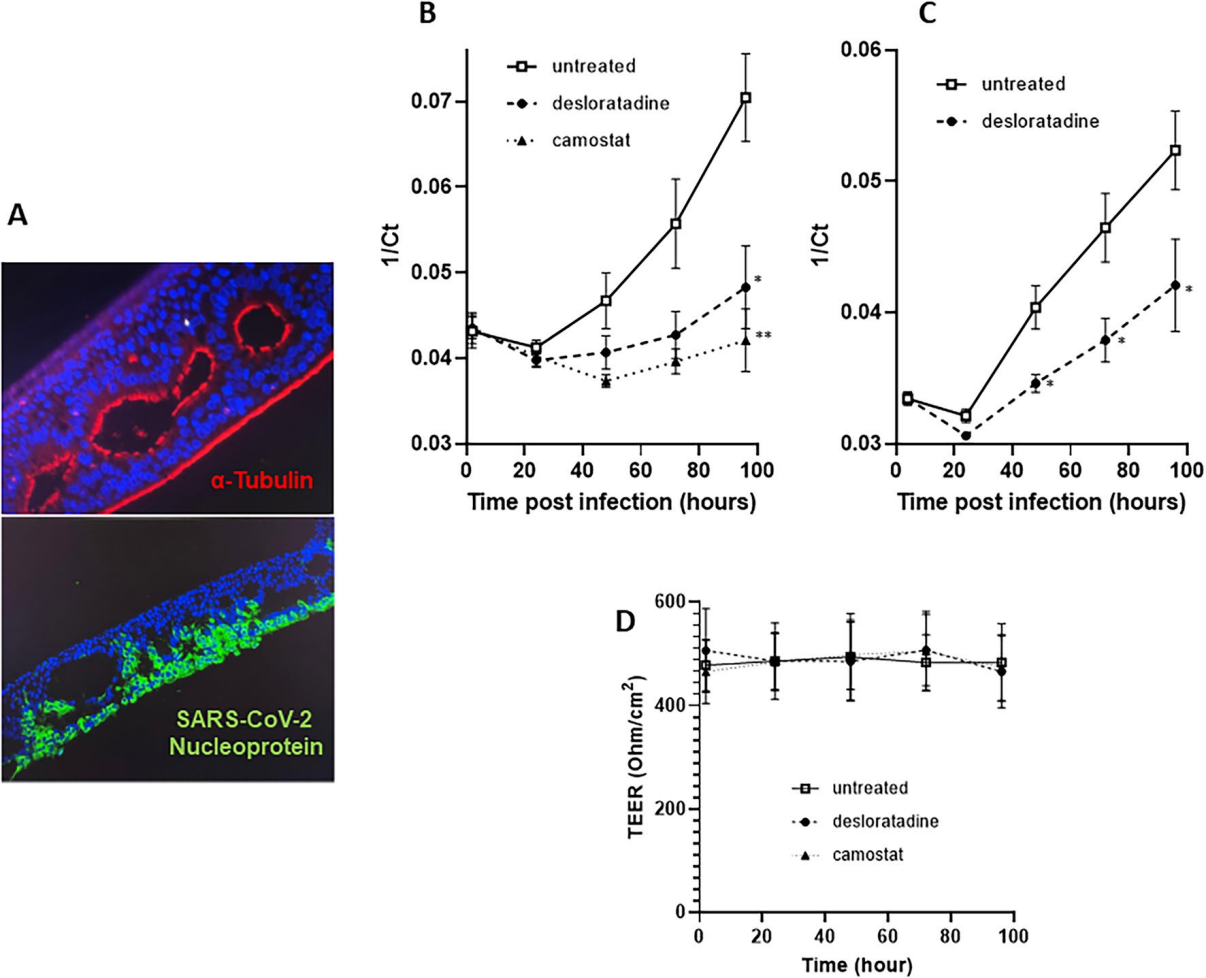
SARS-CoV-2 infection and effect of desloratadine and camostat on SARS-CoV-2 RNA production in primary HNECs. (**A**) Representative images of cross-sectional views of primary human nasal epithelium ciliated cells expressing α-tubulin (red) and the SARS-CoV-2 nucleoprotein (green). Nuclei were stained with DAPI. (**B**) Effect of desloratadine and camostat on dynamics of SARS-CoV-2 RNA production at the apical side of HNECs from three different patients. Results were expressed as the mean of the 1/Ct values ± SEM of the three patients. (**C**) Effect of desloratadine on dynamics of SARS-CoV-2 RNA production at the apical side of HNECs from a healthy donor pool. Results were expressed as the mean of the 1/Ct values ± SEM of two independents experiments. Epithelial integrity was monitored by measuring the transepithelial electrical resistance (TEER) at all-time points for all three patients. Each point represents the average TEER for the three patients untreated, treated with 5 µM desloratadine, treated with 10 µM camostat (**D**). *p < 0.05, **p < 0.01 (Mann‐Whitney *U* test *versus* 1/Ct values of untreated cells).

SARS-CoV-2 infection was monitored by immunohistochemistry staining using an antibody directed against the viral nucleoprotein (Fig. 5A). SARS-CoV-2 nucleoprotein expression was restricted to the apical side of the nasal epithelium, while ciliated cells which express tubulin were the main targets of infection.

The dynamics of the antiviral effect of 5 µM desloratadine was evaluated both on HNECs obtained from 3 independent patients or from a pool of healthy donor (Fig. 5B,C, respectively). The effect of 10 µM camostat (Fig. 5B) was also evaluated as a control in HNECs obtained from the same 3 independent patients. The nasal epithelium was infected with SARS-CoV-2 at its apical pole. After removal of the inoculum, virus production at the apical pole was monitored 4, 24, 48, 72, and 96 h post-infection. Epithelium integrity was assessed by measuring transepithelial electrical resistance (TEER) at all-time points (Fig. 5D). In samples derived from the three patients, delayed virus production was observed with camostat and desloratadine without epithelium alteration, indicating that SARS-CoV-2 infection is inhibited by both drugs. Camostat induced a more pronounced and rapid delay of viral RNA production than desloratadine. Desloratadine effect was confirmed with HNEC obtained from a healthy donor pool (Fig. 5B). Overall, these results suggest that both cysteine protease inhibitors, such as camostat, and lysosomotropic agents, such as desloratadine, can be used to limit SARS-CoV-2 infection of human nasal epithelium cells.

## Discussion

Coronaviruses are enveloped single-stranded positive RNA viruses that represent a serious threat to public health. Indeed, although often asymptomatic or associated with moderate symptoms, SARS-CoV-2 infection can evolve into life-threatening pneumonia and systemic disease^25^. Vaccines have made it possible to slow the course of the SARS-CoV-2 pandemic. Nevertheless, the availability of therapeutic strategies using readily available drugs could be useful to prevent the onset of complications and the need for oxygen therapy in some patients, especially in non-vaccinated individuals and in the case of propagation of vaccine-escape variants, while representing an efficacious tool to prevent the emergence of new coronaviruses in the future.

Coronavirus entry into host cells is an important determinant of viral infectivity, tropism and pathogenesis and represents therefore an interesting target for antiviral therapy. After binding to their receptor, coronaviruses deliver their nucleocapsid into the host cell by fusion of their envelope with the host cell membrane. Cleavage of spike proteins by host cell proteases is essential for this fusion process to happen^26^. It has been shown that SARS-CoV and SARS-CoV-2 preferentially use the cell surface serine protease TMPRSS2 for priming and entry. Nevertheless, the endosome-resident cysteine proteases cathepsin can also be used as alternatives for this process^15,16,27^. Recently, Zhao et al. reported the crucial role of CatL in SARS-CoV-2 infection in humans and humanized mice^17^. Thus, depending on the host protease recruited, SARS-CoV-2 can fuse directly with the plasma membrane or enter cells by endocytosis and fuse with the endosome membrane to deliver its nucleocapsid.

Camostat and nafamostat, two broad-spectrum serine protease inhibitors, clinically approved for other applications in Japan, have been shown to block SARS-CoV-2 fusion at the plasma membrane^15^. E64D, a broad-spectrum cysteine protease inhibitor, has been reported to inhibit SARS-CoV-2 pseudovirion entry trough the endosomal pathway. Furthermore, a synergistic effect of combined camostat and E64D results in a complete blockade of SARS-CoV-2 entry into cells^28–30^. Although it has been shown that, in human airway epithelial cells, coronaviruses generally use surface proteases for entry^31–33^, it appears to be relevant to target both pathways to make sure that a complete blockade of SARS-CoV-2 entry is achieved.

CADs are a large group of drugs with common structural features, including a hydrophobic ring and a hydrophilic side chain containing an ionizable amine. CADs chemical properties lead to their accumulation in acidic intracellular compartments, such as endosomes and lysosomes. The unprotonated neutral CADs are rather hydrophobic and can readily diffuse through the limiting membrane of acidic organelles. In an acidic environment, the basic amine groups of CADs are protonated^34^. The drugs are then trapped inside the lysosomes, leading to their several 100-fold accumulation^35,36^. This uptake mechanism, called lysosomotropism, induces various physiological and morphological alterations of the endolysosomal compartment, which are reversible after treatment discontinuation. Thus, CADs, which include numerous drugs FDA-approved for a wide range of human diseases^6^, could represent a valuable group of repurposed compounds against coronaviruses in general, and notably against SARS-CoV-2.

In the present study, the hypothesis that lysosomotropic compounds could have antiviral activity against SARS-CoV-2 has been tested in Vero-E6 cells with 8 FDA-approved CADs. Five of these CADs, including 2 antiallergics (loratadine and desloratadine), 1 antipsychotic (chlorpromazine) and 2 antidepressants (imipramine and amitriptyline) significantly reduced SARS-CoV-2 RNA production. These results suggest that the antiviral activity of these CADs is related to their lysosomotropic activity rather than to their respective pharmacological targets, a result in keeping with recent observations^37^. In our screening, loratadine and its metabolite desloratadine were the most potent SARS-CoV-2 inhibitors. Both compounds are second-generation FDA-approved antihistaminics that are widely used to treat symptoms associated with seasonal respiratory allergies, urticaria, angioedema or atopic dermatitis.

Cell lines commonly used to perform viral assays exhibit varying levels of TMPRSS2 and cathepsin B/L expression, resulting in the selection by the virus of a preferential entry route. SARS-CoV-2 enters Vero-E6 cells, which do not express high levels of TMPRSS2, via the endosomal pathway. In contrast, SARS-CoV-2 enters Calu-3 cells, which express TMPRSS2, via plasma membrane fusion^24^. In our experiments, desloratadine targeted an early step of the SARS-CoV-2 lifecycle in Vero-E6 cells, whereas it was ineffective in Calu-3 cells, suggesting that desloratidine blocks the endosomal entry route. Because they are lysosomotropic drugs, CADs could indirectly affect the activity of endosome/lysosome-resident enzymes required for the viral lifecycle. Interestingly, it was recently reported that functional inhibition of acid sphingomyelinase, an inner membrane-anchored lysosomal glycoprotein, by the lysosomotropic antidepressant amitriptyline prevented the uptake of SARS-CoV-2 by epithelial cells^38^.

Immortalized cell cultures offer a simple and cost‐effective platform for the investigation of cell biology mechanisms. However, this model suffers from important caveats as far as therapeutic relevance is concerned. We therefore investigated desloratadine antiviral effectiveness in the more relevant primary HNEC polarized culture model, organized, like in the nasal cavity, with an apical side oriented towards the air and a basal side towards the culture medium, therefore mimicking the in vivo conditions. SARS-CoV-2 infection is thought to begin in the nasal epithelium^39^, which expresses high levels of ACE2 receptors^40^. Recently, nasal multiciliated epithelial cells have been shown to be the primary targets of SARS-CoV-2 replication in early-stage COVID-19. Therefore, targeting nasal ciliated cells during the early steps of the SARS-CoV-2 lifecycle may be an appropriate strategy to overcome SARS-CoV-2 propagation, which is transmitted by respiratory aerosols or droplets. Kinetic evaluation of the antiviral properties of camostat and desloratadine in infected HNECs revealed that both drugs reduce SARS-CoV-2 RNA production at the apical pole, indicating that both cysteine protease inhibitors and lysosomotropic agents slow down SARS-CoV-2 replication. However, a more pronounced effect was observed with camostat than with desloratadine, suggesting that SARS-CoV-2 enters HNECs preferentially through TMPRSS2 priming. Recently, Tummino et al*.* evaluated the antiviral effectiveness of the CADs amiodarone and sertraline in a murine model of COVID-19 and observed no significant effect on lung viral titers. However, cellular hallmarks of phospholipidosis were also not observed, suggesting that the lack of antiviral effect in mice lungs could have been related to the absence of lung lysosomal disorders induced by amiodarone and sertraline in their model. Neither sertraline nor amiodarone possess a tricyclic ring, shown in the present study to be a crucial determinant of CADs antiviral activity.

Since the beginning of the COVID-19 pandemic, a potential antiviral effect of lysosomotropic agents has been supported by clinical observations in patients receiving CAD-containing treatments. Indeed, several psychiatric hospitals over the world have observed a lower prevalence of symptomatic infections in psychiatric patients, putting forward the hypothesis of a protective effect of psychotropic treatments^41,42^. Chlorpromazine, a phenothiazine derivative widely used in clinical practice for the treatment of acute and chronic psychoses, was found to have antiviral activity against SARS-CoV-2 in Vero-E6 cells and human A549-ACE2 cells^43^. Nevertheless, chlorpromazine would be inappropriate for systemic prophylaxis of infection in healthy individuals. Fluvoxamine is another approved drug with a molecular structure suggesting lysosomotropism that has been recently tested in a randomized preliminary clinical trial showing a statistically significant reduction of the likelihood of COVID-19 clinical deterioration in patients receiving the compound. No fluvoxamine-treated patients met criteria for clinical deterioration as defined in the study, whereas 8.3% of patients taking placebo met this end point^44^. Hydroxychloroquine, a drug approved for the treatment of malaria, has also been shown to be active in vitro against coronaviruses, including SARS-CoV-2^11^, but little efficacy has been observed in subsequent clinical trials. The disappointing results of the CAD hydroxychloroquine contrast with the encouraging clinical data of chlorpromazine and fluvoxamine. Although all of them have lysosomotropism in common, they differ in their drug profile, which could explain their different effectiveness. Interestingly, chlorpromazine, fluvoxamine and desloratadine are all functional inhibitors of acid sphingomyelinase (FIASMAs), and an association has been reported between FIASMA treatment and a reduced risk of intubation or death in individuals hospitalized for severe COVID-19^45^. Antidepressant exposure was also associated with a reduced incidence of emergency department visitation or hospital admission among SARS-CoV-2 positive patients^46^. An antihistaminic drug such as desloratadine, that has a lysosomotropic effect and inhibits the generation and release of inflammatory mediators and cytokines^18^, would have a clear advantage, compared to antipsychotics and antidepressants, in antiviral therapy. Thus, whether desloratadine could provide a substantial contribution to prophylaxis and systemic therapy of COVID-19 must now be explored in randomized clinical trials.

## Supplementary Information

Supplementary Figures.

## Data Availability

The datasets used and/or analysed during the current study available from the corresponding author on reasonable request.
