# Supplementary material for: Desloratadine, an FDA-approved cationic amphiphilic drug, inhibits SARS-CoV-2 infection in cell culture and primary human nasal epithelial cells by blocking viral entry
Source: Sci Rep. 2022 Dec 6;12:21053. doi: 10.1038/s41598-022-25399-5 (PMC9726831; doi:10.1038/s41598-022-25399-5)

Title

Desloratadine, an FDA-approved cationic amphiphilic drug, inhibits SARS-CoV-2 infection in cell culture and primary human nasal epithelial cells by blocking viral entry

Authors

Margot Morin-Dewaele[1*], Sophie Bartier[1,2,3,4*], François Berry[1*], Rozenn Brillet[1], Dennis Salomón López-Molina[1], Công Trung Nguyễn[1,5], Pascale Maille[5], Kevin Sereno[5], Quentin Nevers[6], Laurent Softic[7], Jean-Marie Vaugeois[8], Bruno Louis[,1,4], Emilie Bequignon[1,2,3,4], Patrice Bruscella[1], André Coste[1,2,3,4], Jean-Michel Pawlotsky[1,9#], Stéphane Jamain[1,10#] and Abdelhakim Ahmed-Belkacem[1#§]

Supplementary Figure 1. Chemical structures of CADs

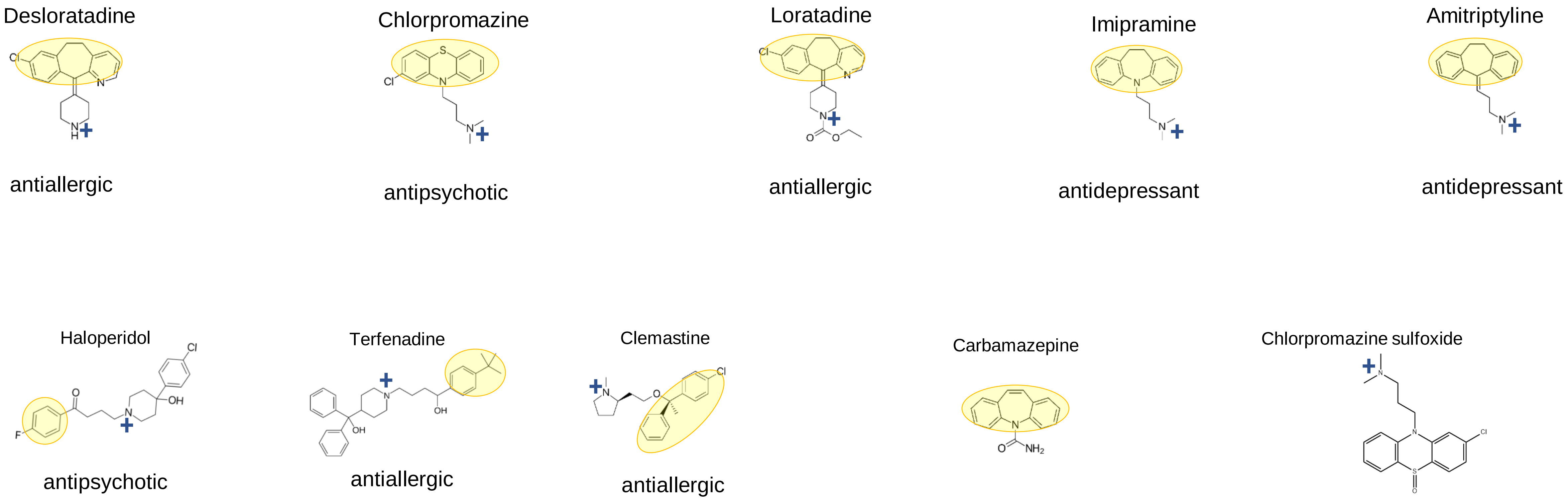

Supplement: Supplementary file 1 — Supplementary Figures. [file 41598_2022_25399_MOESM1_ESM.pdf]
